# A Case of Disseminated Alveolar Echinococcosis Mimicking Metastatic Malignant Disease with Cerebral Involvement

**DOI:** 10.1590/0037-8682-0284-2022

**Published:** 2022-09-30

**Authors:** Ismet Mirac Cakir, Tumay Bekci, Uluhan Eryuruk

**Affiliations:** 1 Giresun University, Faculty of Medicine, Department of Radiology, Giresun, Turkey.

**FIGURE 1:**
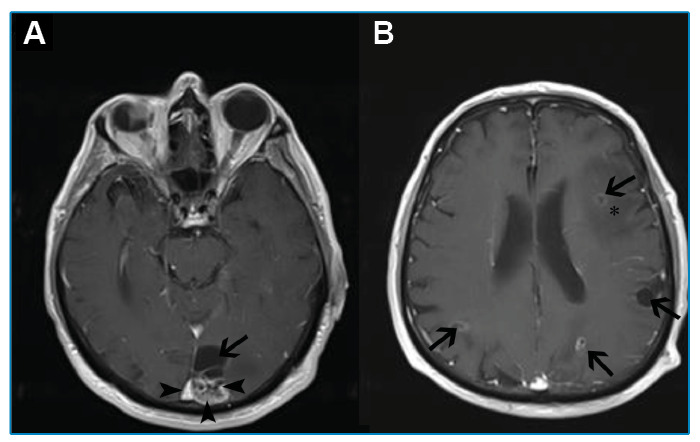
**A.** Axial post-contrast T1-weighted images show heterogeneous enhancement of the mural component (arrowheads) and irregular annular enchancement of the cyst wall in the occipital lobe lesion (arrow). **B.** Axial post-contrast T1-weighted images show multiple bilateral lesions (arrows) with surrounding edema (asterisks).

**FIGURE 2:**
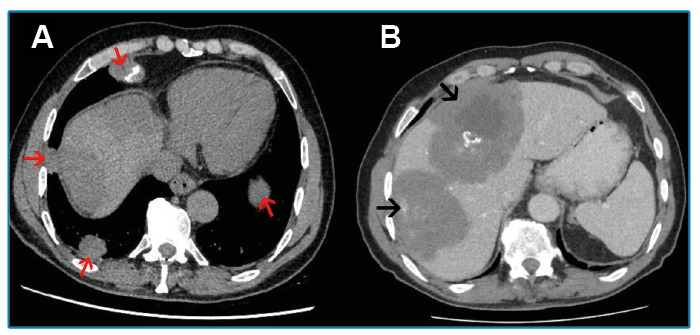
**A.** Thorax CT showed non-cavitary nodular lesions (red arrows) in both lungs, some of which had calcifications. **B.** Contrast-enhanced abdominal CT showed heterogeneous lesions with irregular contours (arrows) containing calcifications and cystic components.

A 68-year-old male patient with no known history of chronic disease or trauma presented with complaints of progressive headache, dizziness, and weight loss that had persisted for six months. Cranial magnetic resonance imaging revealed multiple hyperintense lesions on T2-weighted images with a large area of edema. The lesions showed irregular annular enhancement after administration of an intravenous contrast agent (Figure 1A-B). Based on these findings, cystic metastasis was considered, and thoracoabdominal computed tomography (CT) was performed to investigate the primary malignancy. The thoracic CT showed nodular lesions in both lungs, some of which had calcifications (Figure 2A). On the abdominal CT (Figure 2B), heterogeneous lesions with irregular contours containing calcifications and cystic components were observed. The pathologic examination of the liver lesions revealed germinative membranes of *Echinococcus multilocularis*, and the final diagnosis was alveolar echinococcosis. The patient was administered lifelong medical treatment with albendazole. The cerebral lesions regressed significantly during the first six months of treatment. 

Alveolar echinococcosis is an endemic parasitic disease in many parts of the world and can be fatal if not diagnosed and treated early^1^. Imaging modalities play an important role in diagnosis. Cerebral involvement is rare, and ist incidence is reported in the literature^2^ as < 3%. As in our case, concurrent liver, lung, and brain involvement with malignant imaging features may mimic metastatic malignancy. Clinicians should consider alveolar echinococcosis in the differential diagnosis of disseminated cystic disease, especially in endemic regions.
